# Molecular Dynamics Simulation of 2-Benzimidazolyl-Urea with DPPC Lipid Membrane and Comparison with a Copper(II) Complex Derivative

**DOI:** 10.3390/membranes11100743

**Published:** 2021-09-28

**Authors:** Georgios Rossos, Sotiris K. Hadjikakou, Nikolaos Kourkoumelis

**Affiliations:** 1 Department of Medical Physics, School of Health Sciences, University of Ioannina, 45110 Ioannina, Greece; g.rwssos@gmail.com; 2 Inorganic Chemistry Laboratory, Department of Chemistry, University of Ioannina, 45110 Ioannina, Greece; shadjika@uoi.gr

**Keywords:** benzimidazole derivatives, DPPC, molecular dynamics, permeability

## Abstract

Benzimidazole derivatives have gained attention recently due to their wide pharmacological activity acting as anti-inflammatory, hypotensive, analgesic, and anti-aggregatory agents. They are also common ligands in transition metal coordination chemistry, forming complex compounds with enhanced biological properties, especially in targeted cancer therapy. A key issue to understand anti-tumour effects is drug permeability through cellular membranes, as poor permeability outcomes can avert further futile drug development. In this work, we conducted atomistic molecular dynamics (MD) simulations and biased MD simulations to explore the interactions of 2-benzimidazolyl-urea with a phospholipid bilayer (dipalmitoylphosphatidylcholine, DPPC) together with a previously synthesized copper(II) complex compound. The aim was to study the permeability of these compounds by assessing their free energy profile along the bilayer normal. The simulations indicated that both the ligand (2-benzimidazolyl-urea, BZIMU) and the complex show a similar behaviour, yielding high energy barriers for the permeation process. However, with increasing concentration of BZIMU, the molecules tend to aggregate and form a cluster, leading to the formation of a pore. Clustering and pore formation can possibly explain the previously observed cytotoxicity of the BZIMU molecule via membrane damage.

## 1. Introduction

Transition metal ions are key factors in many biological processes, playing a diverse role in the cell structure, polarization, and function, mostly as prosthetic groups in metalloproteins. Furthermore, metal ions are essential for the regulation and function of several processes associated with membranes. The accumulation of cations on the membrane surface modulates and possibly alters locally its physical properties. It is known that transition elements utilize transporters of different structural and functional characteristics compared to alkali ions [1].

Copper is a vital trace element linked to many biological pathways in aerobic organisms and involved in many metalloenzymes as a structural or catalytic cofactor [2,3]. Copper-based complexes have been studied due to their therapeutic potential especially as anticancer drugs. As copper uptake by cancer cells is higher than that of normal cells, it can suppress tumour growth and progression [4]. Copper (II)-based antitumor drug candidates have been reported as inducers of apoptosis through cellular injury and permeabilization of mitochondrial membrane. For example, a copper(II) complex that is lipophilic (triphenylphosphine, TPP) was reported to induce mitochondrial apoptosis in vitro [5].

The benzimidazole ring is one of the most commonly found moieties in nature among the heterocyclic pharmacophores [6], and several bioactive compounds with the benzimidazole ring having varied structure and activity, acting as a fungicide, anti-parasitic, anti-ulcerative, anti-hypertensive, anti-viral, anti-cancer, and anti-emetic compounds [7]. Benzimidazole derivatives have been utilized as drug scaffolds in medicinal chemistry [8,9,10]. Moreover, they can act as β tubulin inhibitors, suppressing the proliferation of human cancer cells [11]. Urea derivatives, however, show antiproliferative activity against adenocarcinoma cell lines due to their antioxidant activity and their ability to inhibit lipid peroxidation [8,9,12]. Benzimidazole-ureas inhibit vascular endothelial growth factor and tyrosine kinase, preventing angiogenesis, which is crucial for tumour growth [9,13]. Therefore, they act as low-molecular-weight dual inhibitors that are expected to ignore normal proliferative cells, unlike standard cytotoxic chemotherapy. Moreover the efficacy of Cu(II) complexes as chemotherapeutics is reliant on their cytotoxic action and their selectivity towards tumour cells [5]. Hence, the study of the biological activity of the conjugate of benzimdazole with urea and its copper complex is expected to shed light on their mechanism of antiproliferative activity towards efficient drug development.

The preference of molecules that contain the benzimidazole ring refers to the lipophilic microenvironments that aid the transfer of the metal complex to the lipid-water biomolecular interface. The lipophilicity of a molecule is related to its ability to penetrate cell membranes by means of passive diffusion due to the difference of the potential gradient across the membrane, and it has been reported for both organic molecules and metal complexes [14]. Specifically for cisplatin, which is the most widely used anti-tumour agent, it is suggested that at least 50% of drug uptake is due to passive diffusion as the only known method of transport through the membrane in active form [15]. The distribution coefficient mainly depends on hydrophobicity. Nevertheless, a potential drug must be hydrophobic enough to partition into the lipid bilayer, but at the same time not too hydrophobic in order to partition out the lipid bilayer again. Therefore, drug partitioning in the membrane is important in determining whether a drug can be administrated and reflects the relative solubility of the drug in the lipid bilayers of a cell membrane and the aqueous environment. Although cellular accumulation does not determine biological activity, the fine-tuning of lipophilicity can affect the degree of protein binding, cellular uptake, tissue distribution, bioavailability, and toxicity [16].

**Figure 1:**
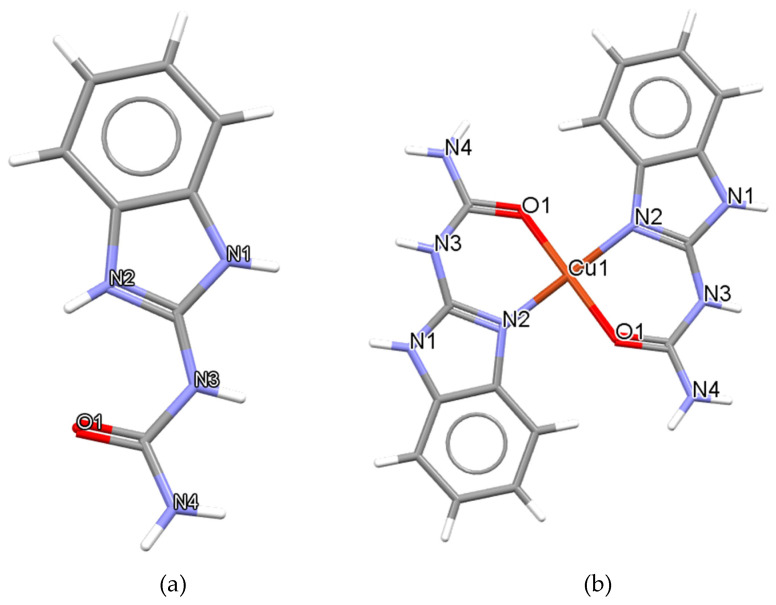
(**a**) BZIMU molecule, (**b**) Cu(II) complex containing two molecules of BZIMU (structures are deposited in the Cambridge Crystallographic Data Centre; CCDC 1517018 and CCDC 1517019).

Two recently synthesized metal-based compounds of the urea derivative, 2-benzimidazolyl-urea (BZIMU) (Figure 1a), with the formulae [Ag(TPP)_2_(BZIMU)NO_3_] and {[Cu(BZIMU)_2_](NO_3_)_2_} (Figure 1b), have been tested by our group for their in vitro antiproliferative activity against human adenocarcinoma cells [8,17], exhibiting significant cytotoxic potential.

The interaction of a drug with lipids is a major factor for its pharmacological activity. Thus, to gain further insight on the interaction of copper(II) and the influence of the ligand to the cell membrane, the model lipids system of DPPC (dipalmitoyl-phosphatidyl-choline) were chosen in this work. DPPC is a zwitterionic lipid that simulates the bulk lipid of eukaryotic membranes [18]. Therefore, from a biological perspective, the DPPC is considered as the membrane model of mammalian cells. The membrane of eukaryotic cells consists almost exclusively of zwitterionic lipids such as phosphatidyl-choline and appears uncharged.

In this study, we applied atomistic molecular dynamics (MD) simulations to study the interaction of a copper(II) complex with BZIMU (Cu-complex) and of BZIMU molecule with the DPPC lipid bilayer model. Our aim was to compare the diffusion of the molecules into the membranes by evaluating the location and agglomeration in each studied system. Therefore, we also assessed the effect of the concentration of BZIMU on the partition coefficient into the model membranes.

## 2. Computational Methods

We applied a combined approach to study the interaction of each molecule with the lipid bilayer model. First, we performed an MD simulation without constraints, with the molecules placed in the polar part of each bilayer. This simulation was analysed to understand where the studied molecule prefers to settle and how it affects the properties of each bilayer. Next, we used the umbrella sampling [19] method to create the potential drug location distributions by pushing along the axis of the bilayer. This method provided the free energy profile indicating the energy boundaries along the movement path, and we analysed the dynamical and structural alterations during this process. Finally, we studied the dynamical behaviour of multiple (fifteen) molecules (15BZIMU) randomly distributed in the water–membrane interface to evaluate the effect of concentration on the distribution into the membrane using the z-constraint method [20].

The DPPC lipid bilayer of 128 lipids units was modelled using the GROMOS-53A6 force field with a modified version of parameter 53A6 to include Berger lipids. Full hydration settings were used with ~30:1 water/lipid ratios. The simulation box was of average dimensions of 6.8 nm in x-direction, 6.8 nm in y-direction for all the systems, and 5.4 nm in z-direction for the DPPC-BZIMU and DPPC-Complex systems, including 6.4 nm for the DPPC-15BZIMU system of the water layer due to periodic boundary conditions.

The Dundee PRODRG server [21], based on the GROMOS force field, was used to generate the necessary topology files and coordinates as input files from the GROMACS computer package [22]. Particle Mesh Ewald electrostatics with a real-space Coulomb cut-off of 1.4 nm was used with van der Waals interactions cut-off at 1.4 nm. Bonds were constrained using the LINCS algorithm. Partial charges for the drug–molecules were computed using the Hartree–Fock method and the 6-31G* basis set. The water molecules were modelled by the TIP3P water model, providing compatibility with the parameters of lipid molecules. Partial density was evaluated with the GROMACS *gmx density* subroutine which computes the density as a function of slices along the *z*-axis, producing a density histogram binning relative to the centre in absolute box coordinates.

MD simulations were performed at the atomic level to examine the difference between the compounds BZIMU and its complex with copper while crossing through the cell membrane. The free energy profile during the transfer of a molecule in each case was estimated by calculating the potential of mean force (PMF). Generally, PMF is calculated with biased MD simulations which include either umbrella sampling or z-constraint method [20]. In this study, PMF was calculated using the umbrella sampling method for the permeability study and a z-constraint for the evaluation of the concentration of the BZIMU molecule on the partition study. In the former case, each of two molecules was placed in the polar part of the membrane. It was then pushed to move to the centre of each bilayer along the *z*-axis using the umbrella sampling method. A harmonic potential of 500 kJ/mol/nm^2^ was applied to the molecule at a rate of 1.5 nm/ns for the distance between the centres of mass of the molecule and the lipid bilayer. Approximately ten configurations were then selected for each system, representing different positions on the *z*-axis as the molecule moved from one polar region of the bilayer to the other. Each configuration was simulated for 2 ns at equilibrium with the NPT ensemble, using the Parinello–Rahman barostat, and for 10 ns for the MD. Each molecule was constrained to specific positions along the *z*-axis but was free to move in the xy plane. The WHAM [23]-weighted histogram analysis method was used to estimate the free energy profile of the molecules in each lipid bilayer, ensuring that the molecule distributions of adjacent windows sufficiently overlapped. To examine the effect of the concentration, we inserted 15 molecules of BZIMU at the polar part of the DPPC membrane. We then applied a harmonic potential of 500 kJ/mol/nm^2^ to observe the accumulation site, as well as the efficiency of the accumulation in the form of clustering.

### 2.1. Lateral Diffusion Coefficient

Lateral diffusion depends on the nearby molecules. It is a fast and spontaneous movement relative to the translational movements between the two layers. Ensemble averaged mean–square displacement (MSD) was evaluated for the bilayer systems to assess lipid diffusion. The average MSD for the DPPC molecule was calculated with the *gmx msd* subroutine of the GROMACS software. Diffusion coefficients are calculated from time– and ensemble–mean squared displacement of the chosen molecular species by restarting the MSD computation at even time intervals across the trajectory. The linear part of the MSD curves, (i.e., the MSD-t plot) was fitted to determine D_α_. The α parameter, which characterizes the diffusion mechanism [24], was obtained from a non-linear fit to the general form of the equation for the MSD by assuming that the diffusion of the lipids progresses in two dimensions:

(1) $$W\left(t\right)≅4D_{\alpha }t^{a}\;\;\;\;\;\;0<a<2\;\;\;\;\;\;\;\;\;$$

Normal diffusion corresponds to a linear time dependence and α = 1, while anomalous diffusion resembles non-linear dependence of the MSD vs. time. Values of α between 0 and 1 characterize sub-diffusion and above 1, super-diffusion. Typically, the motion of lipids in membranes is considered as sub-diffusive or normal with transient sub-diffusion [25].

The resulting MSD curve exhibits good averaging at short time intervals while becoming gradually worse at longer simulation times. Here, the full trajectories were used by restarting the MSD calculation every 50 ps. For the lipids movement, the MSD, W(t) = <(Δr(t))^2^> = <(r(t + t_0_) − r(t_0_))^2^>, is typically used to calculate the fractional self-diffusion coefficient:

(2) $$D_{\alpha }=\frac{1}{2n_{f}}\underset{t\to \infty \;}{\lim}\frac{<{\left(\Delta r\left(t\right)\right)}^{2}>}{t}$$

where n_f_ represents the number of the translational degrees of freedom, the r(t) is the position of the centre of mass (COM) of the simulated molecules at time t, Δr(t) is the deviation of the r(t_0_) and r(t + t_0_) position vectors of the simulated molecules at time t_0_ and t + t_0_ respectively, and the brackets denote ensemble average (i.e., mean squared deviation of the position vectors at time t_0_). Although biological systems are characterized by time length scales far beyond current atomistic simulations (t tends to infinity in Equation (2)), time remains an elusive parameter in MD simulations due to the finite computational resources. In order to test whether a simulation is long enough, we can test if the molecules explore a sufficiently diverse region of configuration space by calculating the MSD and comparing the outcome to the simulation box length (see Computational Methods). As permeation is a slow process requiring several nanoseconds, we followed standard simulation procedures to obtain statistically significant information such as to either drag the molecules into the membrane or to place them within the membrane to start with. These processes ensured sampling the membrane–penetrant interactions across the whole simulation space during the given simulation time scale.

### 2.2. Umbrella Sampling

The umbrella sampling [19] is a popular method to assess the translocation free energy profile of compounds in that the relative free energy of different states can be evaluated relative to the reaction coordinates (ξ). In its general application, multiple windows of the initial structures at different values of the reaction coordinates are produced. A pulling potential of the form:

(3) $$E_{\mathrm{bias}}=E_{\mathrm{unbias}}+\omega_{i}\left(\xi \right)$$

is applied to every window, where i indicates the corresponding window during the umbrella sampling, according to a harmonic pulling function:

(4) $$\omega_{i}\left(\xi \right)=\frac{1}{2}K{\left(\xi -\xi_{i}\right)}^{2}$$

where $\omega_{i}\left(\xi \right)$ is the potential energy for the restraint of the ith simulation window, K is the force constant, and ξ_i_ is the reference point. During the simulations, when the system escapes the reaction coordinate, a pulling potential is added to restore the system to its initial state. When the distance between two groups changes, the system deviates from equilibrium, as work is offered to the system. The difference in free energy between two distances of simulated states that are not anymore equilibrated is described by the Jarzynski’s equation:

(5) $${\Delta G}_{\mathrm{AB}}=-k_{B}\mathrm{Tlog}{<e^{-{\beta W}_{\mathrm{AB}}}>}_{A}$$

where W_AB_ is the work along the path from state A to state B, the brackets denote the average of the canonical ensemble of the initial state A, and β = 1/k_B_T, where k_B_ is the Boltzmann’s constant and T is the temperature. Lateral diffusion increases with temperature; here, simulation experiments were performed at physiological temperature, 310 K, which also preserved the fluidity of the membrane.

PMF is the potential that derives by integrating the mean force over a total of conformations. The free energy profile can be calculated by the following equation:

ΔG(z) = −RT ln P(z) + U(z)(6)

where P(z) and U(z) are the distribution of distance and biasing potential along the bilayer normal, respectively.

## 3. Results and Discussion

**Figure 2:**
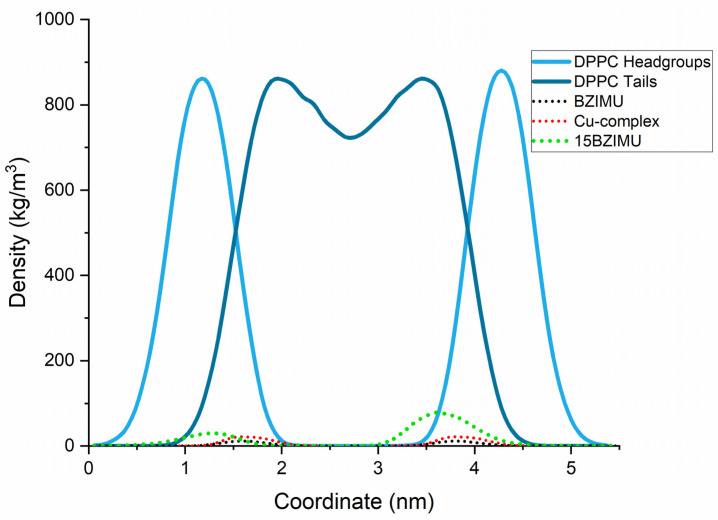
Partial density profiles along the bilayer normal (*z*-axis). 15BZIMU indicates the presence of fifteen BZIMU molecules.

To validate the bilayer construction protocol, the partial density profile of the systems was plotted (Figure 2). As the membrane is a complicated system to simulate, the correct fluidity was tested by plotting the partial density profile of the system. The density profile concurs with the published experimental [26] and simulation data [27], suggesting that the system was normally equilibrated. The thickness of the membrane was approximately 4 nm and agrees with the literature. The distinctive low at the centre of the bilayer due to the disorder of the lipid tails was also present. The distance over which the headgroup density drops from 90% to 10% of the maximum value is the interfacial width. Similar to typical simulations of fully hydrated DPPC, this value was less than 1 nm, implying minimal perpendicular lipid motion and water penetration.

**Figure 3:**
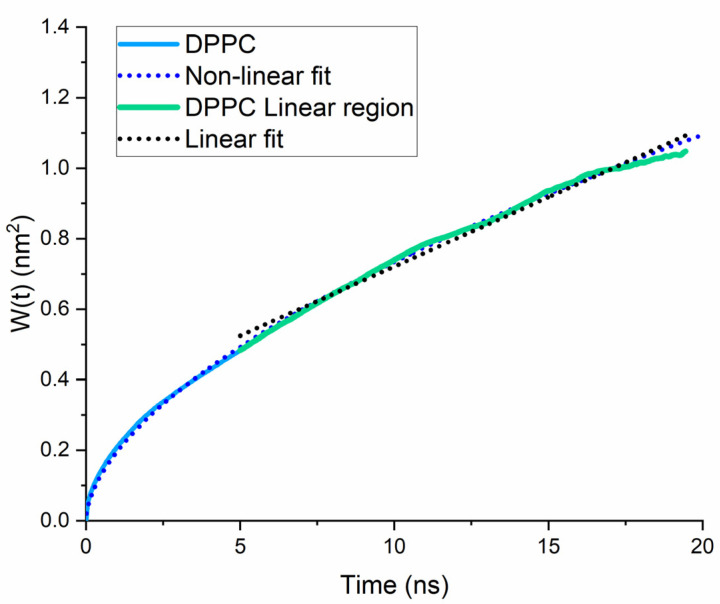
Simulated molecule-averaged MSD for DPPC lipids bilayers. The non-linear fit resulted from fitting to Equation (2).

Figure 2 illustrates that both BZIMU and Cu-complex are located into the bilayer near the lipid head groups that represent the dense area of the membrane. Lipid lateral dynamics for DPPC was analysed by plotting the MSD (Figure 3) and by calculating the diffusion coefficient from the corresponding MD simulations. The non-linear fit yields α = 0.57 which indicates a transient sub-diffusion. The coefficient of diffusion was calculated to D_α_ = 0.039 × 10^−5^ cm^2^/s which is in excellent agreement with the reported value [28,29].

**Figure 4:**
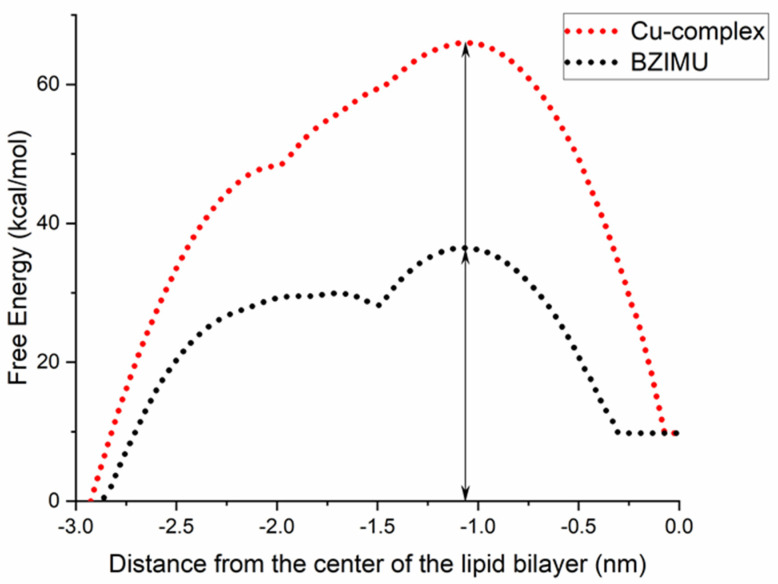
Free energy profiles for Cu-complex and BZIMU. The vertical line shows the maximum energy barrier (ΔG^(pen)^). Profiles are accepted as symmetrical across the centre of the bilayer as also shown in Figure 2.

The free energy profiles for Cu-complex and BZIMU are shown in Figure 4. Typically, the free energy is set to zero in bulk water (i.e., z < −2.8 nm). Both compounds showed an initial stabilizing interaction (in the range of ~3–5 kcal/mol) upon leaving the bulk solvent and entering the headgroup region of the membrane (~2 nm from the centre). Two free energy barriers can be detected in Figure 4 with the first at z ~1 nm, where the density reaches its maximum value. When the polar molecules are inserted into the bilayer, the free energy increases, as the movement is less favourable due to the hydrophobic lipid tails at the middle. The reverse procedure is apparent at the bilayer centre having the minimum density and represents the second energy barrier. The first minimum in Figure 4 is at 1.5 and 1.8 nm for BZIMU and Cu-complex, respectively, which is attributed to the thermally accessible region of RT energy barrier (i.e., 0.6 kcal/mol at 323 K). The sharp dip of the energy in the centre of the bilayer is attributed to the hydrogen bond break between the molecules and the solvent. Overall, for both molecules, there is a high energy permeation barrier at ΔG^(pen)^ = 65.9 and 36.4 kcal/mol for Cu-complex and BZIMU, respectively.

**Figure 5:**
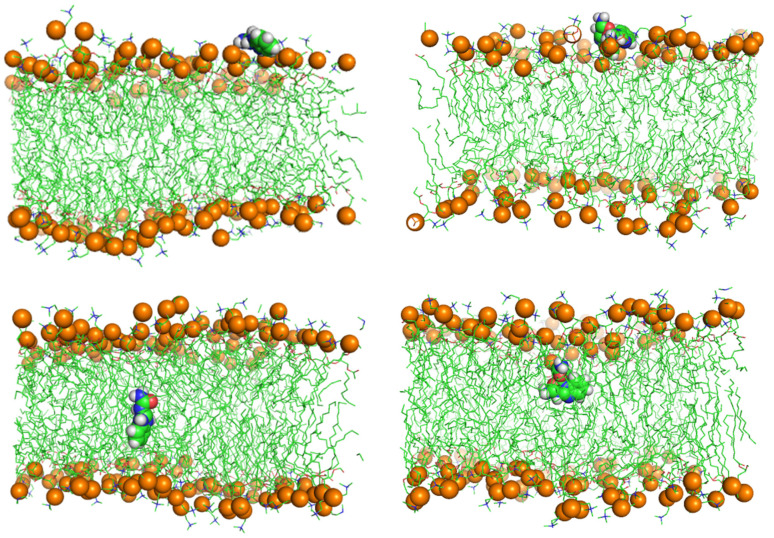

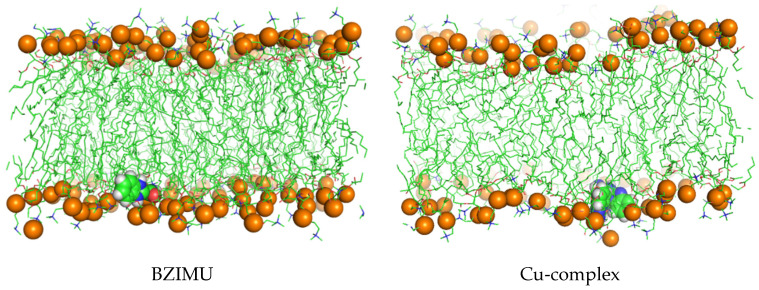
Trajectory of BZIMU and Cu-complex entering the membrane. Snapshots taken at 2, 3, and 4 ns for BZIMU and at 1.5, 1.8, and 3 ns for Cu-complex.

Figure 5 shows screenshots of the trajectories of the BZIMU and Cu-complex using MD umbrella sampling.

From the results, we concluded that it is energetically unfavourable for BZIMU and Cu-complex to pass through a DPPC membrane. Although the partition of a molecule depends on the relative energy, the permeability depends on the free energy barrier and the PMF method can predict permeability. Specifically in this case, PMF clarifies a long-standing question on whether similar transition complexes, acting as potential drugs, can overcome that energetic obstacle during the transmembrane passing or if they exert their action as biologically active metal ions. We argue that metal complexes like Cu-complex and the corresponding derivatives of urea, probably disrupt the membrane rather than passively penetrate it.

### BZIMU Cluster Formation

To examine the effect of the BZIMU concentration on the permeation of the molecules, we increased the solute concentration to fifteen BZIMU. We then applied a harmonic potential along the *z*-axis.

**Figure 6:**
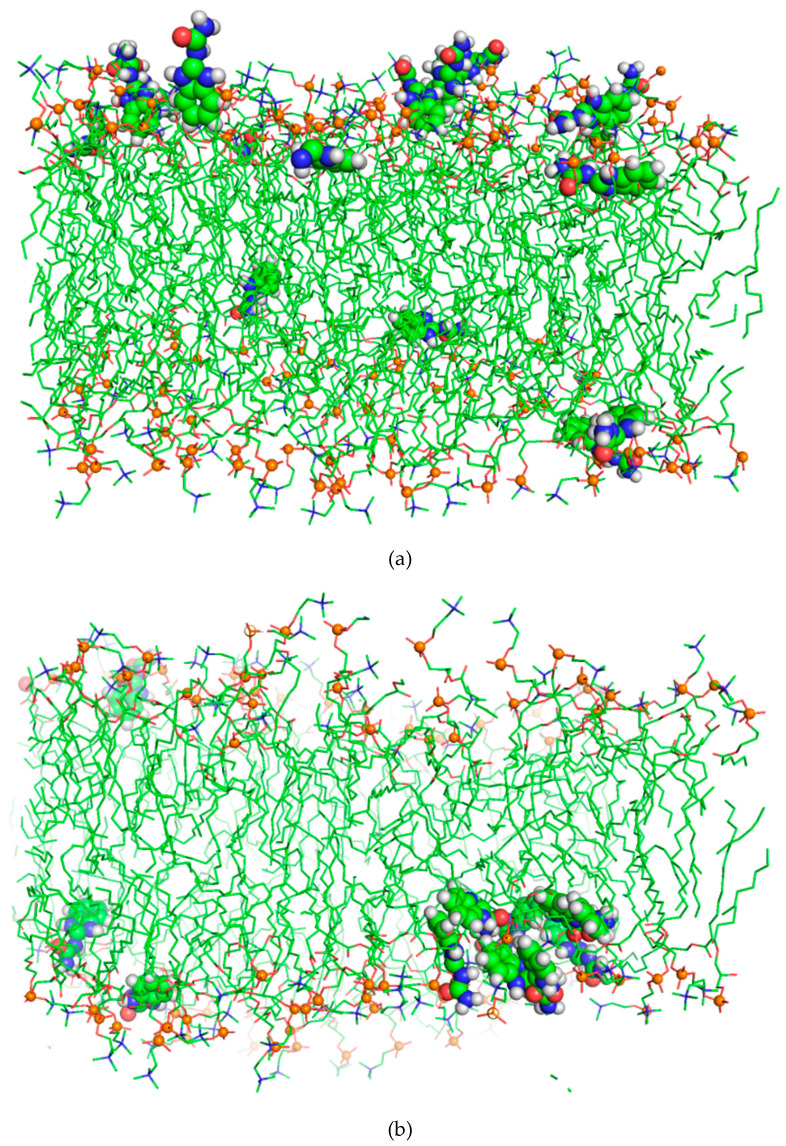
Cluster formation of BZIMU molecules under the effect of a *z*-axis potential. Snapshots taken at 0.3 (**a**), and 7.4 ns (**b**).

As shown in Figure 6, the BZIMU (15BZIMU) molecules rapidly (t = 7 ns) form a cluster near the centre of the bilayer, with only four BZIMU molecules away from the cluster. Similar behaviour has been also reported in an analogous MD study regarding the permeation of bisphenol-A through DPPC. The mass density profile (Figure 2) shifted towards the centre compared to the single BZIMU in the membrane, implying a low lipid solubility despite the hydrophobic character of the molecule.

**Figure 7:**
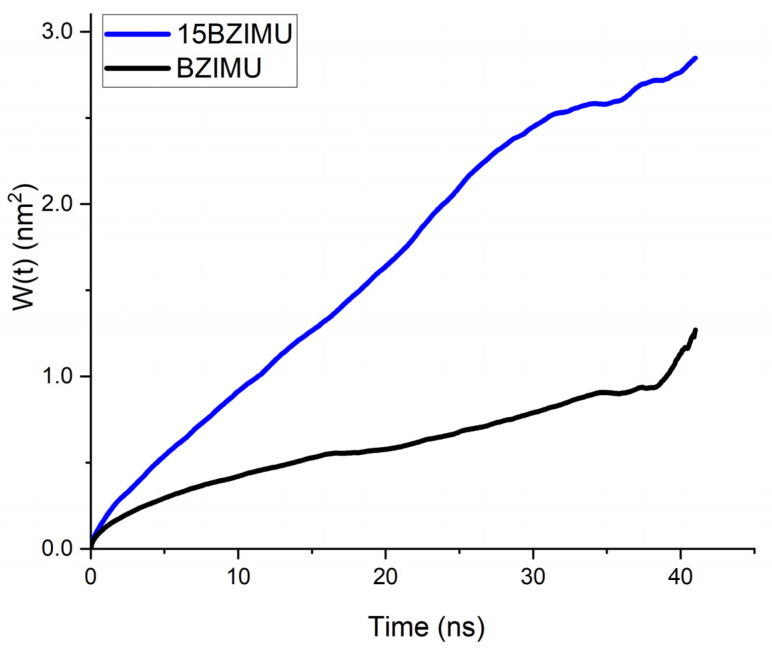
Simulated MSD for a single molecule of BZIMU and the BZIMU cluster of 15 molecules.

The lateral diffusion coefficient of BZIMU increases when 15 molecules are present in the bilayer (Figure 7). The values obtained by linear fit were 0.019 × 10^−5^ cm^2^/s and 0.074 × 10^−5^ cm^2^/s for BZIMU and 15BZIMU, respectively. Although these values are not quantitatively accurate, they can be qualitatively compared. Therefore, the diffusion coefficient increases with the number of molecules. As a result, the diffusion of lipids in the DPPC membrane decreases since 15 BZIMU molecules occupy additional space during their insertion as the translational mobility of the lipids correlates with the permeability capacity of small molecules across the membrane [30].

**Figure 8:**
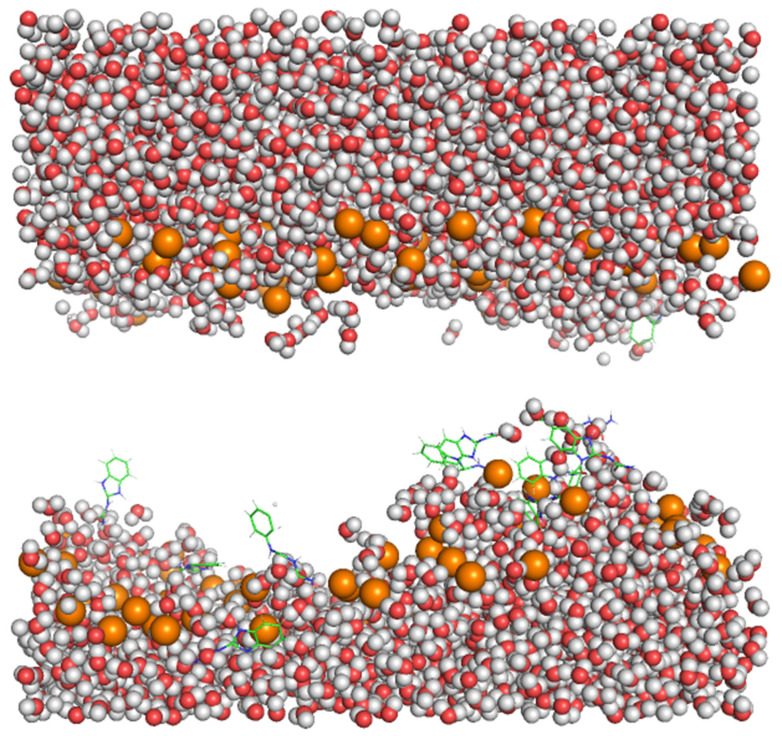
Pore formation. BZIMU molecules form a cluster that is surrounded by water molecules.

The formation of the BZIMU cluster leads to a pore formation (Figure 8) similar to the study by Chen et al. [31]. The pore fills with water molecules while the lipid structures are moved away from the bilayer. This internal disruption is due to the strong dispersion interactions between the sp2 carbon atoms of the BZIMU molecule and the lipid tails [31,32]. Furthermore, membrane fluidity plays a major role in the mechanism of passive diffusion. To function properly, cell membranes must retain fluidity to permit molecular transport into or out of the cell [33]. It was recently suggested that decreased membrane fluidity may affect cisplatin transport into cells while being the main cause of the resistance mechanism and inefficient uptake by target cells [34]. The fluidity of the bilayers is also dependent on the inclusion of molecules into the bilayer as well as on the possible interactions with the lipids tails as described. In our case, during the pore formation, the structural rigidity of the bilayer is disturbed, and water molecules fill the hole by flowing into the membrane, increasing its fluidity. Therefore, increased concentration of drug-like molecules in the membrane seems to affect the diffusion and uptake efficacy. Thus, the pore formation may provide clues on the previously found cytotoxicity of these compounds by damaging the cell membrane through disruption. Nevertheless, to comprehend direct membrane effects in in vitro systems, further studies should be conducted that demonstrate the ability of the potential agents to induce ion pores (in electrophysiology membrane current measurements), perhaps as a result of direct membrane (i.e., lipids) binding [35,36].

## 4. Conclusions

Transition metal complexes based on copper have shown significant therapeutic potential. However, the precise mechanism of their action is still under investigation. For the successful passive diffusion, metal complexes are typically decorated with mild lipophilic molecules in order to penetrate the cell membrane. Here, we studied the translocation and aggregation of BZIMU along with the translocation of an analogous copper(II) complex. We found that the energy barrier for the two molecules prevent them from readily diffusing into the membrane. Previous studies showed significant in vitro activity of the studied molecules. Our results indicate that this cytotoxic activity is exerted by disrupting the membrane integrity. High concentration of the BZIMU ligand may be the key parameter that leads to cluster formation, which in turn, can possibly yield a certain cytotoxic potential by the formation of a pore. Further experimental work is needed, aimed at proving the ability of the molecules to induce transient or stabilized pores, to validate the theoretical findings proposed in this work.

## Data Availability

All results can be replicated by following the described methodology.
